# Genotype-Specific Rhizosphere Microbiome Assembly Mediates Biochar-Induced Salt Tolerance in Sorghum

**DOI:** 10.3390/cimb48020186

**Published:** 2026-02-06

**Authors:** Yingying Xu, Lingyu Zhang, Zhichang Gao, Zhijian Shi, Peng Li, Ruitao Xu, Jianghui Cui

**Affiliations:** College of Agronomy, Hebei Agricultural University, Baoding 071000, China; yingyingxu1019@163.com (Y.X.); zhanglingyu07@163.com (L.Z.); gzc11486@163.com (Z.G.); shizhijian1982@126.com (Z.S.); lipeng@hebau.edu.cn (P.L.); xrt_welcomeyou@163.com (R.X.)

**Keywords:** carbon amendment, crop adaptation, microbial community assembly, plant–microbe interaction, rhizosphere microbiome, salt tolerance, precision agriculture

## Abstract

Sorghum genotypes differentially shape their rhizosphere microbiomes to cope with salt stress; however, the modulatory role of biochar in this genotype-specific plant–microbe interplay remains unclear. In this study, we investigated how salt-sensitive (Henong 16, HN16) and salt-tolerant (Jizaonuo 1, JZN) sorghum genotypes leverage biochar to assemble distinct functional rhizosphere microbiomes under salt stress (5 g kg^−1^ NaCl). Biochar application (20 g kg^−1^) alleviated ionic stress by reducing soil electrical conductivity (EC decreased by 46% in HN16) and enhanced soil fertility through increased organic matter (SOM increased by 26% in JZN). 16S rRNA gene sequencing revealed that biochar selectively enriched genotype-specific, stress-resistant taxa. The salt-sensitive HN16 primarily recruited *Sporosarcina* (a genus reported to exhibit salt tolerance and nitrogen-fixing capabilities) and Intrasporangiaceae, thereby rapidly establishing a rhizosphere barrier. In contrast, the salt-tolerant JZN consistently enriched *Salinimicrobium* (an extreme halophile) and the family LWQ8, forming more complex and stable co-occurrence networks with a higher proportion of positive correlations (81%). Plant genotype was the primary determinant of core microbiome assembly: *Bacillus* and *Arthrobacter* dominated in HN16, whereas *Sphingomonas* and *Streptomyces* prevailed in JZN. Biochar reinforced this genotype-specific assembly by modulating soil pH and SOM, which were identified as key drivers of microbial community divergence. Importantly, these biochar-shaped microbial modules showed significant positive correlations with increased plant biomass. Our findings demonstrate that biochar enhances salt tolerance not merely by improving soil properties, but primarily by facilitating the deterministic assembly of genotype-specific, functional rhizosphere microbiomes. This mechanistic insight shifts the paradigm of biochar from a universal soil amendment to a precision tool for rhizosphere engineering, providing a genotype-aware foundation for enhancing salinity resilience in sustainable agriculture.

## 1. Introduction

Soil salinization is a major abiotic threat to global crop production, particularly in arid and semi-arid regions. It severely restricts crop growth and yield formation by inducing osmotic stress, ion toxicity, and nutritional imbalance [1,2,3]. Sorghum, an important C4 crop valued as food, feed, and bioenergy feedstock, plays a crucial role in the agricultural systems of these regions due to its strong drought tolerance and adaptability [4,5]. However, sorghum growth is still significantly inhibited by salt stress [6]. Therefore, exploring effective strategies for ameliorating saline soils is essential for ensuring stable sorghum production.

In recent years, biochar, a carbon-rich material produced by pyrolysis of biomass under oxygen-limited conditions, has shown great potential in remediating saline soils. Its mechanisms of action are multifaceted. Beyond the direct adsorption of Na^+^ via its large specific surface area and high porosity—for instance, reducing soil electrical conductivity (EC) by up to 50% in some studies [7]—biochar application regulates soil physicochemical properties in ways that collectively foster a more favorable rhizosphere microenvironment. For example, it can buffer soil pH towards a neutral range, mitigate compaction to increase porosity by 10–20%, and significantly elevate soil organic matter (SOM) content, which serves as a key energy source for soil biota [8,9]. The rhizosphere microbial community, whose activity and composition are directly influenced by these improved soil conditions, acts as a bridge between plants and soil, playing a central role in nutrient cycling, stress resistance, and plant health maintenance [10,11,12,13].

Salt stress significantly reduces soil microbial diversity and disrupts community structure, while biochar application can reverse these negative effects by providing physical refuge and chemical energy sources for microorganisms [14,15,16]. Concurrently, emerging evidence highlights the pivotal role of plant genetics in structuring rhizosphere microbiomes [17] and the intricate chemical dialogue that governs plant–microbe interactions [18]. Moreover, biochar-induced shifts in soil microbial communities have been linked to improvements in soil fertility and ecosystem multifunctionality [19,20]. However, most studies have focused on the singular effects of biochar on either soil properties or microbial communities in a generic context. A systematic understanding of how biochar and plant genotype conjointly and dynamically drive the assembly of a functional, stress-resilient rhizosphere microbiome under salt stress remains elusive [21].

Different crop genotypes may respond differentially to biochar through their unique rhizosphere microecosystems [22]. This genotype-specific regulatory pathway is a key determinant of biochar’s field efficacy, yet its underlying mechanisms—particularly the temporal dynamics of microbial recruitment and the formation of genotype-specific cooperative networks—are poorly understood. While root exudate-mediated chemical communication is a primary mechanism through which plants shape their rhizosphere [17], how biochar modulates this genotype-dependent dialogue to recruit stress-resilient microbiomes under salt stress remains unclear. Such knowledge gaps limit our ability to predict and optimize biochar application in genotype-specific cropping systems under saline conditions.

Therefore, this study aims to elucidate the genotype-specific mechanisms through which biochar enhances salt tolerance in sorghum by mediating rhizosphere microbiome assembly. We hypothesize that biochar differentially shapes the rhizosphere microbial communities of salt-sensitive (HN16) and salt-tolerant (JZN) sorghum genotypes, leading to distinct functional networks that enhance plant resilience under salt stress. Specifically, we propose that: (1) biochar will recruit genotype-specific, stress-resistant microbial taxa; (2) these taxa will form more cooperative and stable co-occurrence networks in the salt-tolerant genotype; and (3) the biochar-driven shifts in soil properties (e.g., pH, SOM) will be key drivers of microbial community differentiation and plant growth promotion. Through an integrated approach combining soil physicochemical analysis, high-throughput 16S rRNA sequencing, and microbial network analysis, this study seeks to provide a mechanistic basis for precision biochar application in saline agriculture, guided by plant genotype.

## 2. Materials and Methods

### 2.1. Experimental Materials

A total of 185 sorghum accessions, including conventional germplasm, landraces, and recombinant inbred lines (RILs), were provided by the Sorghum Breeding Laboratory of Hebei Agricultural University (the full list is provided in Table A1, Appendix A).Based on preliminary salt tolerance screening (evaluated by relative germination rate, seedling survival rate, and biomass under salt stress), the salt-sensitive cultivar Henong 16 (HN16) and the salt-tolerant cultivar Jizaonuo 1 (JZN) were selected for a full-growth-period pot experiment in a greenhouse at Hebei Agricultural University. The biochar, supplied by Hebei Badu Biotechnology Co., Ltd. (Baoding, Hebei Province, China), was produced from corn cobs via high-temperature pyrolysis under oxygen-limited conditions. Its key physicochemical properties were as follows: iodine adsorption value, 992 mg/g; moisture content, 8.7%; ash content, 7.5%; pH, 8.3; total carbon, 83.1%; and total nitrogen, 1.3%.

The soil used in this study was collected from the top layer (0–20 cm) of an agricultural field at the Hebei Agricultural University experimental station in Baoding, China. The soil is classified as a sandy loam (Typic Haplustalf) with the following basic properties before any amendment: total nitrogen 0.55%, alkali-hydrolyzable nitrogen 505.1 mg kg^−1^, available phosphorus 24.4 mg kg^−1^, available potassium 345 mg kg^−1^, soil organic matter (SOM) 12.57 g kg^−1^, pH 6.54, and electrical conductivity (EC) 25.1 mS cm^−1^. The soil was air-dried, sieved through a 2-mm mesh, and mixed with vermiculite in a 1:1 ratio (*v*/*v*) to form the cultivation substrate.

### 2.2. Experimental Design

A pot experiment was conducted with two sorghum cultivars: the salt-sensitive HN16 and the salt-tolerant JZN. Three treatments were established: CK group (normal soil control), T2 group (5 g kg^−1^ NaCl salt stress treatment), and T1 group (5 g kg^−1^ NaCl salt stress with 20 g kg^−1^ biochar application). Each treatment had three replicates (Table 1). The pot culture experiment used pots of 30 cm × 21 cm, each filled with 10 kg of the cultivation substrate (see Section 2.1). Salt-contaminated soil with a salt content of 5 g kg^−1^ was prepared by thoroughly mixing NaCl with the soil, and the salt content was verified using an EC meter. Rhizosphere soil samples were collected at five growth stages: before sowing (S0), seedling stage (S1), jointing stage (S2), heading and flowering stage (S3), and filling and maturation stage (S4). Basic soil physicochemical properties were measured using standard methods: soil water content by the gravimetric method, pH with a pH meter, EC with a conductivity meter (model DDS-307A, Shanghai Yidian Scientific Instrument Co., Ltd. [Shanghai, China]), and SOM content by the potassium dichromate heated acid digestion method. Total soil microbial DNA was extracted using the FastDNA^®^ SPIN Kit for Soil (MP Biomedicals, USA).

### 2.3. Soil Physicochemical Analysis

Soil pH was measured using a pH meter at a water-to-soil ratio of 2.5:1 (*w*/*v*). Soil electrical conductivity (EC) was measured using a conductivity meter(model DDS-307A, Shanghai Yidian Scientific Instrument Co., Ltd. [Shanghai, China]) at a water-to-soil ratio of 5:1 (*w*/*v*). Soil organic matter (SOM) was determined by the potassium dichromate volumetric method, as described in the third edition of Soil and Agricultural Chemistry Analysis [23]. Soil bulk density (SBD) was measured using the core method, and soil particle density was determined via the water displacement method. Soil porosity (SP) was calculated using the following formula: SP (%) = (1 − SBD/particle density) × 100%.

### 2.4. Microbial Community Analysis

Total soil microbial DNA was extracted from rhizosphere soil samples using a commercial soil DNA extraction kit (FastDNA^®^ SPIN Kit for Soil, MP Biomedicals, [Santa Ana, CA, USA]). The V3–V4 hypervariable regions of the bacterial 16S rRNA gene were amplified using the universal primers 338F (5′-ACTCCTACGGGAGGCAGCAG-3′) and 806R (5′-GGACTACHVGGGTWTCTAAT-3′) [24]. Amplicons were sequenced on the Illumina NovaSeq 6000 platform (Illumina, Inc. [San Diego, CA, USA]) at Majorbio Bio-Pharm Technology Co., Ltd. [Shanghai, China] using paired-end sequencing. Raw sequences were quality-filtered, denoised, and processed into amplicon sequence variants (ASVs) using DADA2 within the QIIME2 pipeline (version 2020.11) [25,26]. Taxonomic assignment of ASVs was performed against the SILVA 138 database [27]. Alpha diversity indices (ACE, Chao1, Shannon) and beta diversity based on Bray–Curtis dissimilarity were calculated using QIIME2. Principal coordinate analysis (PCoA) and permutational multivariate analysis of variance (PERMANOVA) were conducted using the vegan package in R [28].

Linear discriminant analysis effect size (LEfSe) was employed to identify differentially abundant microbial taxa between groups, with a linear discriminant analysis (LDA) score threshold of >2.0 and a statistical significance level of *p* < 0.05 [29]. Co-occurrence networks were constructed using the Molecular Ecological Network Analysis (MENA) platform (http://ieg4.rccc.ou.edu/mena, accessed on 16 June 2025]) based on Spearman’s correlation coefficients (|r| > 0.6, *p* < 0.01) [30]. Network topological properties, including the number of nodes, edges, and modularity, were calculated and visualized using Gephi software (version 0.10.1, Gephi Consortium [Paris, France]) [31].

### 2.5. Statistical Analysis

Data processing, including the calculation of means, standard deviations, and coefficients of variation (CV), was performed using Microsoft Excel 2010 (Microsoft Corporation [Redmond, WA, USA]). After analysis of variance (ANOVA), Duncan’s multiple range test (*p* < 0.05) was performed as a post hoc test to assess significant differences among treatments. Correlation and cluster analyses were performed and visualized using Origin 2022 (OriginLab Corporation, Northampton, MA, USA). Analysis of variance (ANOVA) (version 2022b, OriginLab Corporation [Northampton, MA, USA]). Analysis of variance (ANOVA) and principal component analysis (PCA) were performed using SPSS 22.0 (IBM Corp. [Armonk, NY, USA]). Redundancy analysis (RDA) and Mantel tests were conducted using the vegan package (version 2.6-4) in R (v4.3.1, R Foundation for Statistical Computing [Vienna, Austria]) to elucidate the relationships among environmental factors, microbial communities, and plant-soil system properties. All microbial data analyses were completed on the Majorbio Cloud Platform (https://www.majorbio.com, accessed on 5 October 2025).

## 3. Results

### 3.1. Biochar Differentially Improves the Rhizosphere Microenvironment in a Genotype-Dependent Manner

Significant differences (*p* < 0.05) were observed in rhizosphere soil pH, EC, porosity, and organic matter content among treatments (CK, T2, T1) across growth stages for both sorghum cultivars.

**Figure 1:**
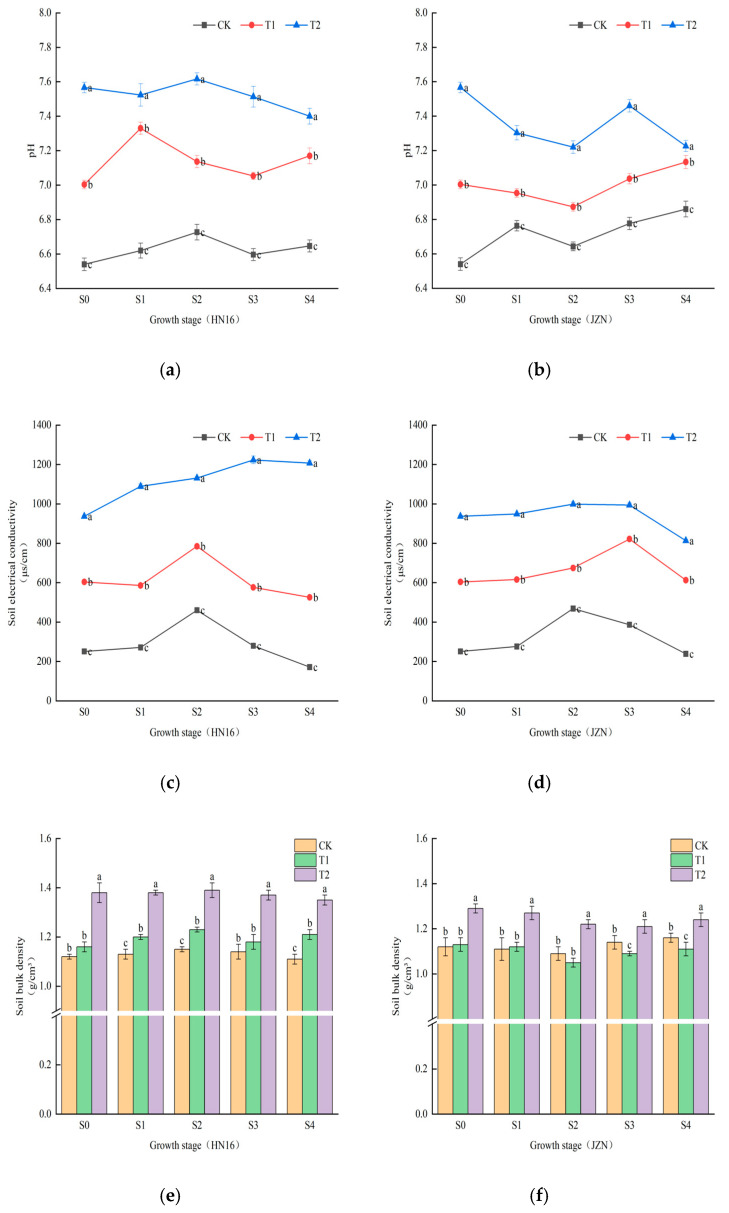

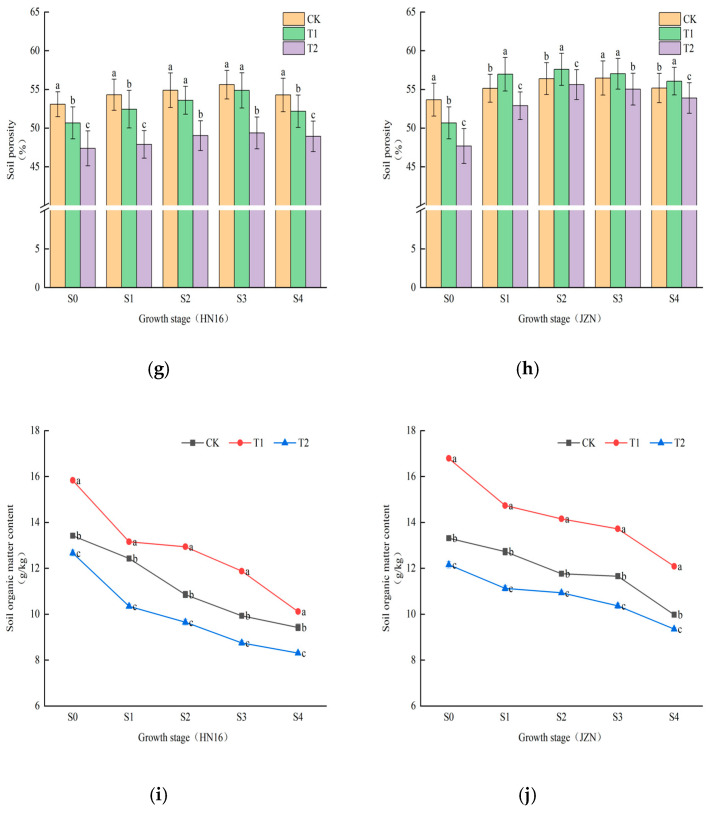
Effects of biochar amendment on the rhizosphere soil physicochemical properties of two sorghum cultivars under salt stress. (**a**,**b**) Soil pH; (**c**,**d**) Soil electrical conductivity (EC); (**e**,**f**) Soil bulk density (SBD); (**g**,**h**) Soil porosity (SP); (**i**,**j**) Soil organic matter (SOM). Error bars represent the standard deviation (SD) of three biological replicates. Different lowercase letters above bars within the same growth stage indicate significant differences among treatments (*p* < 0.05) according to ANOVA followed by Duncan’s multiple range test.

Salt stress (T2) significantly increased the rhizosphere soil pH of both cultivars (Figure 1a,b), indicating that salt introduction caused general soil alkalization. However, biochar (T1) exhibited effective buffering capacity, stabilizing soil pH within a moderate range between CK and T2. At multiple growth stages, the pH value under T1 treatment for the JZN cultivar was slightly lower than that for HN16 at the corresponding stage, suggesting a more sensitive pH regulation response to biochar in JZN, creating a micro-environment closer to neutral. Changes in soil EC values directly confirmed the establishment of a saline environment (Figure 1c,d). Under T2 treatment, EC values of both cultivars increased sharply, particularly more drastically in HN16. T1 treatment significantly reduced the actual rhizosphere soil EC value. At the S1 stage, the EC values of HN16 and JZN under T1 treatment decreased by approximately 46% and 35%, respectively, compared to T2. This indicates that biochar effectively immobilized salt ions through its strong adsorption properties, with a numerically more prominent salt adsorption effect on the HN16 cultivar, thereby alleviating ion stress. T2 treatment increased soil bulk density (SBD, Figure 1e,f), decreased soil porosity (SP, Figure 1g,h), and consequently deteriorated the soil physical structure in both cultivars. As shown in Figure 1g,h (HN16) and (JZN), soil porosity under salt stress (T2) declined progressively across growth stages, reflecting structural degradation. In contrast, biochar amendment (T1) mitigated this decline, with porosity values consistently higher than T2 and, in the case of JZN, even surpassing the control (CK) during key growth stages (S1–S2). The ameliorative effect of T1 for HN16 primarily manifested as alleviating compaction, with its bulk density and porosity indices significantly better than T2 but not surpassing CK. For JZN, T1 demonstrated proactive optimization, with its porosity at stages like S1 and S2 even significantly higher than CK. This indicates that biochar more thoroughly improved the soil structure for JZN, providing superior physical space for root development. Throughout the growth period, T1 treatment consistently maintained the highest soil organic matter levels for both cultivars (Figure 1g,h). This significant fertility enhancement was particularly evident in JZN; at the S0 stage, the organic matter content in its T1 treatment was 26% higher than CK, superior to the 18% increase in HN16.

These results indicate that biochar effectively constructed a rhizosphere microenvironment alleviating salt stress through the integrated pathway of “adsorbing salts, buffering alkalinity, improving structure, and enhancing fertility.” Among these, the HN16 cultivar may benefit more from salt adsorption and alleviation of physical compaction, whereas the JZN cultivar additionally gained synergistic benefits from soil structure optimization and fertility enhancement, providing key soil ecological basis for explaining the differences in their growth responses.

### 3.2. Temporal Dynamics of the Microbial Community in Response to Salt Stress and Biochar Amendment

Analysis of alpha and beta diversity revealed distinct response patterns of the rhizobacterial communities to salt stress and biochar amendment between the two sorghum genotypes.

**Figure 2:**
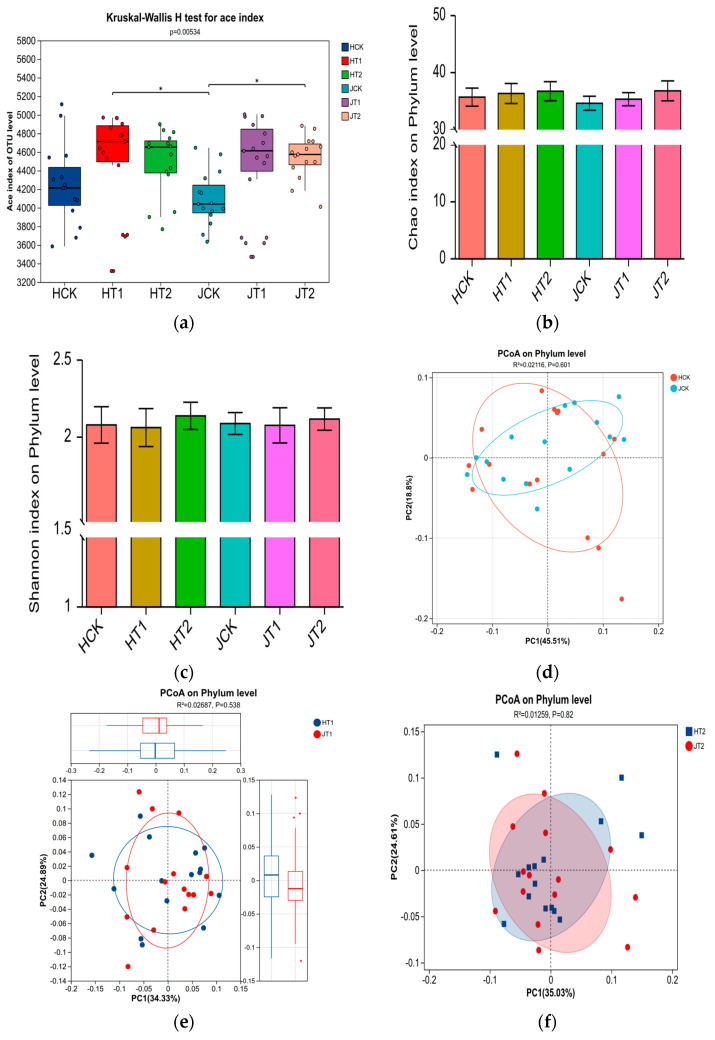
Diversity and structural changes in rhizosphere bacterial communities under salt stress and biochar treatment. (**a**) Effects of treatments on bacterial species richness (ACE index). Bars represent the mean ACE value, and error bars indicate standard deviation (SD) of three biological replicates. (**b**) Chao1 index, corroborating trends in species richness. Bars represent mean Chao1 value with SD error bars. Different lowercase letters above bars indicate significant differences among treatments within the same growth stage (*p* < 0.05). (**c**) Shannon index, indicating community diversity across growth stages. Bars represent mean Shannon index with SD error bars. Different lowercase letters above bars indicate significant differences among treatments within the same growth stage (*p* < 0.05). (**d**) PCoA plot comparing microbial community structures between HCK (HN16 control) and JCK (JZN control) groups (genotype effect under control conditions). Each point represents a sample; shapes distinguish genotypes: circles for HN16, triangles for JZN. PERMANOVA results (R^2^ and *p* values) are indicated; while visual separation is observed, statistical significance was not reached for genotype within each treatment (all *p* > 0.05). (**e**) PCoA plot comparing HT1 (HN16 + biochar) and JT1 (JZN + biochar) groups (genotype effect under biochar amendment). Points are shaped by genotype as in (**d**). (**f**) PCoA plot comparing HT2 (HN16 + salt) and JT2 (JZN + salt) groups (genotype effect under salt stress). Points are shaped by genotype as in (**d**).

Alpha diversity indices (ACE, Chao1, and Shannon) varied significantly among treatments (*p* < 0.05). Salt stress (T2) significantly reduced bacterial species richness compared to the control (CK). Specifically, the ACE index of the HT2 group was approximately 18% lower than HCK, while a more pronounced reduction of 22% was observed in JT2 compared to JCK (Kruskal–Wallis test, *p* < 0.00534; Figure 2a). Biochar amendment (T1) effectively mitigated this decline. The ACE index in the JT1 group recovered to a level comparable to JCK and was significantly higher than in JT2, with a more modest recovery observed in HN16 (HT1 vs. HT2).

The response trend in species richness was corroborated by the Chao1 index (Figure 2b). T2 treatment significantly reduced the Chao1 index (*p* < 0.01), with reductions of approximately 16% in HN16 and 24% in JZN. T1 treatment reversed this decline, particularly in JZN, where the Chao1 index in JT1 returned to a level close to JCK and was significantly higher than in JT2 (*p* < 0.05). Regarding community diversity, the Shannon index dynamics differed between genotypes across growth stages (Figure 2c). In HN16, the Shannon index of HT2 decreased significantly during the later stages (S3–S4). In contrast, HT1 remained relatively stable and was significantly higher than HT2 during S2–S3. JZN exhibited greater stability under biochar treatment; the Shannon index of JT1 remained above 6.8 during key growth stages (S1–S3) and was slightly higher than JCK during S1–S3.

Beta diversity analysis further highlighted genotype-dependent community assembly. Principal coordinate analysis (PCoA) was performed on three paired genotype comparisons under equivalent treatments: HCK vs. JCK, HT1 vs. JT1, and HT2 vs. JT2 (Figure 2d–f). While PERMANOVA did not indicate statistically significant separation between genotypes within each comparison (all *p* > 0.05), visual segregation was observed in the ordination plots. HCK and JCK samples showed the most pronounced separation along PC1 (45.51% of variance). Community structures also remained visually distinct between genotypes in both the HT1 vs. JT1 and HT2 vs. JT2 comparisons.

Collectively, these results demonstrate that salt stress reduced rhizobacterial richness and diversity, with a more severe impact observed in the salt-tolerant genotype JZN. Biochar amendment mitigated these negative effects, with a particularly strong restorative effect on community richness and stability in JZN. Furthermore, microbial community structure was primarily shaped by plant genotype, and this genotypic influence persisted under both salt stress and biochar amendment.

### 3.3. Biochar Drives Genotype-Specific Recruitment of Stress-Resistant Microbial Taxa

**Figure 3:**
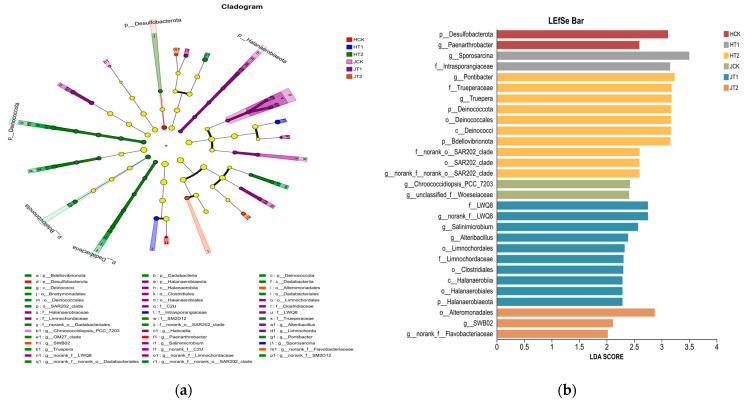
Biochar drives genotype-specific recruitment of beneficial microbial taxa in the sorghum rhizosphere under salt stress. (**a**) Cladogram of discriminative microbial taxa identified by LEfSe analysis across treatments. Nodes are colored according to the treatment group in which the taxa are significantly enriched: [red for HN16_CK, green for HN16_T2, blue for HN16_T1, pink for JZN_CK, purple for JZN_T1, orange for JZN_T2] (**b**) Comparison of LDA scores for discriminative microbial taxa across treatments.

LEfSe analysis revealed significantly enriched taxa (LDA > 2.0, *p* < 0.05) in response to biochar amendment under salt stress, with distinct microbial signatures between HN16 and JZN. In the salt-sensitive genotype HN16 (Figure 3a,b), salt stress (T2) significantly suppressed beneficial taxa, including *Desulfobacterota* (a bacterial phylum) and *Paenarthrobacter* (a genus), while biochar amendment (T1) led to a notable enrichment of *Sporosarcina* (a genus) and *Intrasporangiaceae* (a bacterial family). *Sporosarcina* exhibits strong salt tolerance and nitrogen-fixing capacity, thereby improving nitrogen cycling under saline conditions. In the salt-sensitive genotype HN16, biochar amendment (T1) led to a notable enrichment of *Sporosarcina* and *Intrasporangiaceae*, which may contribute to stress alleviation through nitrogen fixation and secondary metabolite secretion, respectively.

Conversely, under salt stress without biochar (T2), the same genotype showed enrichment of different taxa, including *Pontibacter* and *Deinococcota*. During the growth period, *Sporosarcina* (a genus) and *Intrasporangiaceae* (a bacterial family) in HN16 exhibited higher Linear Discriminant Analysis (LDA) scores (LDA > 2.0) at the early stages (S0–S1), indicating that biochar rapidly established a salt-resistant microbial community shortly after application. As the growth period progressed to S2–S4, the relative abundance of salt-responsive taxa (e.g., *Pontibacter* and *Deinococcota*) gradually increased, reflecting adaptive succession of the rhizosphere microbial community. In the CK treatment, *Desulfobacterota* (a bacterial phylum) and *Paenarthrobacter* (a genus) remained stably enriched throughout all growth stages, which supports normal soil ecological functioning.

The salt-tolerant genotype JZN showed a similar regulatory trend to HN16 but differed in the specific taxa involved. Specifically, the T1 group in JZN was markedly enriched in LWQ8 (a bacterial family) and *Salinimicrobium* (a genus); the latter possesses extreme salt tolerance and may mitigate salt-induced phytotoxicity through metabolic adjustments, while LWQ8 participates in soil nutrient cycling and directly supports plant growth. The T2 group in JZN was enriched in taxa such as *SWBO2* (a genus) and *norank_f_Flavobacteriaceae* (a bacterial family with unclassified rank), which likely help maintain microbial community structure under salt stress through specific physiological metabolism and represent signature taxa for JZN’s response to salinity. In terms of temporal dynamics, LWQ8 and *Salinimicrobium* in the JZN T1 group displayed higher LDA scores (LDA > 2.0) from S0 to S2, indicating that the enriching effect of biochar persisted longer in JZN compared to HN16. In the JZN T2 group, the abundance of taxa such as *SWBO2* increased significantly during the later growth stages (S3–S4), suggesting a delayed adaptive response of the JZN rhizosphere microbiome to salt stress compared to HN16. The dominant taxa in the JZN CK treatment remained consistent with those in HN16, with stable enrichment of *Desulfobacterota* and *Paenarthrobacter*.

In summary, salt stress suppressed beneficial taxa (e.g., *Desulfobacterota* and *Paenarthrobacter*) in both HN16 and JZN genotypes. Biochar counteracted this inhibitory effect by enriching stress-resistant functional taxa—specifically *Sporosarcina* in HN16 and *Salinimicrobium* in JZN—thereby reshaping the rhizosphere microbial community under salt stress. The key taxa in both genotypes exhibited stage-specific succession during the growth period: the biochar-induced enrichment was concentrated in the early growth stages (S0–S1) for HN16, whereas this effect was more sustained (S0–S2) for JZN.

### 3.4. Genotype-Specific Formation of Sorghum Rhizosphere Microbiome and Biochar Regulation Effects

**Figure 4:**
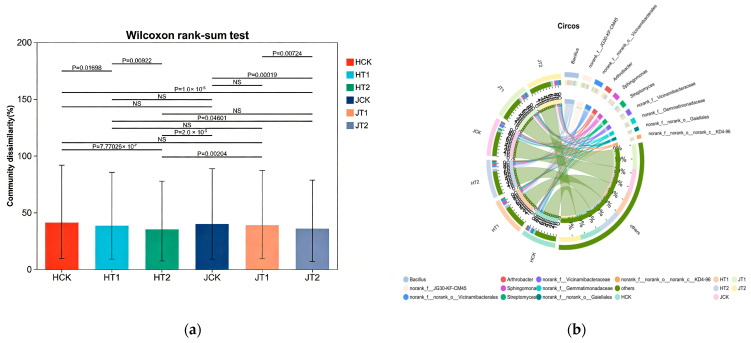
Assembly Patterns of Rhizosphere Microbial Communities in Response to Salt Stress and Biochar Amendment across Sorghum Genotypes. (**a**) Significant differences in microbial community structure among groups based on Bray-Curtis distance. (**b**) Specific associations between key microbial taxa and sample groups.

**Figure 5:**
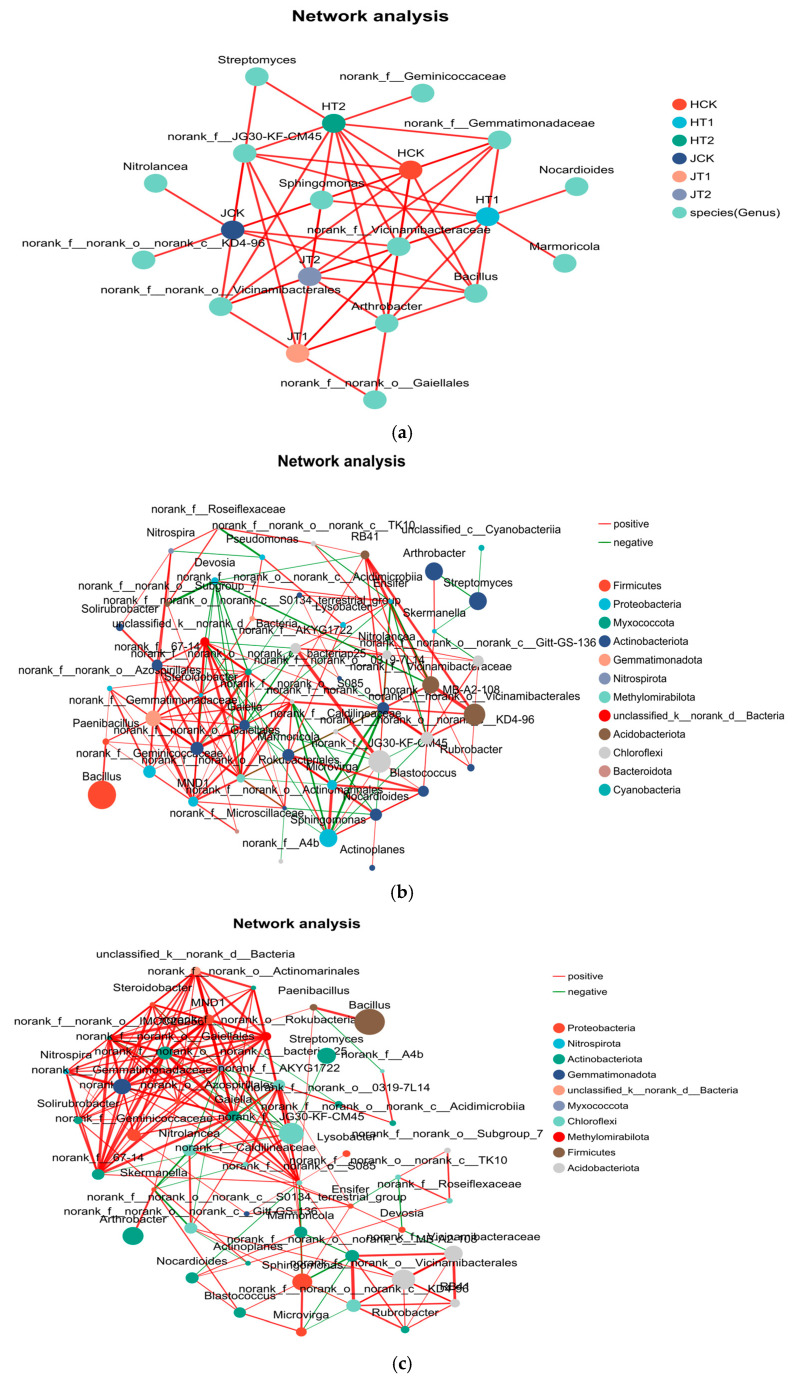
Biochar enhances the complexity and modularity of rhizosphere microbial co-occurrence networks in a genotype-dependent manner. (**a**) OTU-level microbial co-occurrence networks of HN16 and JZN genotypes under different treatments. (**b**) Microbial co-occurrence network at the genus level of the HN16 genotype. (**c**) Microbial co-occurrence network at the genus level of the JZN genotype.

PERMANOVA confirmed that plant genotype was the primary driver of microbial community composition (*p* < 0.001), with biochar and salt stress further shaping the community in a genotype-specific manner. Analysis of community dissimilarity (Figure 4a) revealed significant differences in microbial composition between the HN16 and JZN genotypes across all treatment groups (HCK vs. JCK: *p* = 0.00019; HT1 vs. JT1: *p* = 0.00724), indicating that plant genotype is a key driver of rhizosphere microbial assembly. Within each genotype, significant compositional shifts were also observed among the different treatments. For HN16, notable differences were detected between the HCK and HT2 groups (*p* = 2.0 × 10^−5^), and similarly for JZN between the JCK and JT2 groups (*p* = 4.601 × 10^−2^), demonstrating that both salt stress and biochar amendment further shape the microbial community in a genotype-specific manner. The Circos plot (Figure 4b) visually illustrated the genotype-dependent enrichment of key microbial taxa. The HN16 genotype was predominantly associated with taxa such as *Bacillus* and *Arthrobacter*, which have been frequently linked to salt tolerance and plant growth promotion in previous studies and showed positive correlations with plant biomass under biochar amendment in our system (Figure 5). *Bacillus* showed the strongest association in the HCK group, while *Arthrobacter* was significantly enriched in the HT1 group, indicating their roles as core microorganisms for HN16 in responding to varying conditions. In contrast, the JZN genotype was characterized by the dominance of *Sphingomonas* and *Streptomyces*, with *Sphingomonas* being prominent in the JCK group and *Streptomyces* highly linked to the JT1 group, reflecting a distinct microbial interaction network specific to JZN. Throughout the growth stages, dynamic succession patterns were observed. In HN16, *Bacillus* was predominantly enriched during the early stages (S0–S2), while the relative abundance of *Arthrobacter* increased in later stages (S3–S4), indicating phase-specific microbial assembly. For JZN, *Sphingomonas* was consistently enriched from S0 to S3, and *Streptomyces* increased markedly by the final growth stage (S4), demonstrating a successional rhythm distinct from that of HN16. Notably, T1 modulated microbial assembly in both genotypes. In HN16, the abundance of *Arthrobacter* was significantly higher in the HT1 group than in HCK, whereas in JZN, *Streptomyces* was markedly enriched in JT1 compared to JCK. These results confirm that biochar can selectively enrich beneficial microbial taxa in a genotype-dependent manner.

In summary, HN16 and JZN exhibited distinct genotype-specific microbial assembly patterns. The rhizosphere of HN16 was consistently colonized by salt-tolerant, plant-growth-promoting taxa such as *Bacillus* and *Arthrobacter*, which are crucial for nutrient cycling and stress resistance. In contrast, JZN assembled a microbiome dominated by *Sphingomonas* and *Streptomyces*, which contribute significantly to substance decomposition and secondary metabolite synthesis. These differential assembly patterns underscore the deterministic role of plant genotype in shaping a customized rhizosphere microbiome.

### 3.5. Biochar Fosters Cooperative Microbial Networks with Enhanced Stability in a Genotype-Dependent Manner

Network analysis indicated that biochar significantly increased edge numbers and the proportion of positive correlations in co-occurrence networks (*p* < 0.01), with more pronounced effects in the salt-tolerant genotype JZN. At the OTU level, the T1 treatment promoted the formation of highly dense co-occurrence networks structured around genotype-specific microbial taxa. As illustrated in Figure 5a, the nodes corresponding to the T1 treatment in both the HN16 and JZN genotypes established extensive connections with a broad spectrum of OTUs, exhibiting a significantly wider interaction range than the corresponding nodes in the CK and T2 groups. In HN16, the number of OTUs associated with *Bacillus* increased by approximately 140% under the T1 treatment compared to the CK group. In JZN, the number of OTUs linked to *Sphingomonas* reached 2.3 times that in the CK group. This trend was consistently observed in the genus-level co-occurrence networks (Figure 5b,c), where the T1 treatment significantly increased both the total number of nodes and edges. Specifically, the edge numbers in the HT1 and JT1 groups were 41% and 35% higher than their respective CK groups, indicating that biochar effectively broadened the dimensions of microbial interactions.

Biochar amendment not only increased the connectivity of microbial networks but also qualitatively optimized microbial interactions. As shown in Figure 5b,c, the proportion of edges representing positive correlations was significantly higher in T1 networks than in the CK and T2 groups. Specifically, positive interactions accounted for 78% and 81% of all edges in the HT1 and JT1 groups, respectively, substantially exceeding the proportions observed in their corresponding CK groups. This cooperative interaction pattern facilitated functional complementarity and resource coordination between key taxa—such as *Bacillus* and *Arthrobacter* in HN16, and *Sphingomonas* and *Streptomyces* in JZN—thereby enhancing the structural cohesion and overall buffering capacity of the microbial community against external stress.

Furthermore, T1 treatment promoted the development of a highly modular and stable microbial community structure. The HT1 network differentiated into specialized modules centered on *Bacillus* and *Arthrobacter* (Figure 5b), which reinforced community cohesion through resource complementarity and metabolic cooperation, thereby improving salt stress resilience. These modules—dominated by *Bacillus* (nutrient decomposition) and *Arthrobacter* (stress resistance)—interacted via bridge taxa, enabling efficient material exchange and functional coordination, which collectively enhanced stress resistance efficiency. The JZN genotype exhibited a more pronounced response to biochar amendment. Its T1 network formed four well-defined functional modules (Figure 5c), including those led by *Sphingomonas* and *Streptomyces* responsible for organic degradation and secondary metabolism. These modules were interconnected via the bridge taxon *Nocardioides*, facilitating cross-module metabolite exchange. In contrast, the JCK group displayed a loosely structured network with extensive functional overlap.

In summary, biochar amendment strengthened the structural integrity and stability of the sorghum rhizosphere microbial co-occurrence network under salt stress by expanding interaction dimensions, enhancing positive species cooperation, and promoting functional modularization. Although this effect was observed in both genotypes, it was more pronounced in JZN, further confirming that microbial responses to biochar are genotype-specific.

### 3.6. Biochar-Driven Shifts in Soil Properties and the Rhizosphere Microbiome Are Linked to Enhanced Sorghum Biomass Under Salt Stress

**Figure 6:**
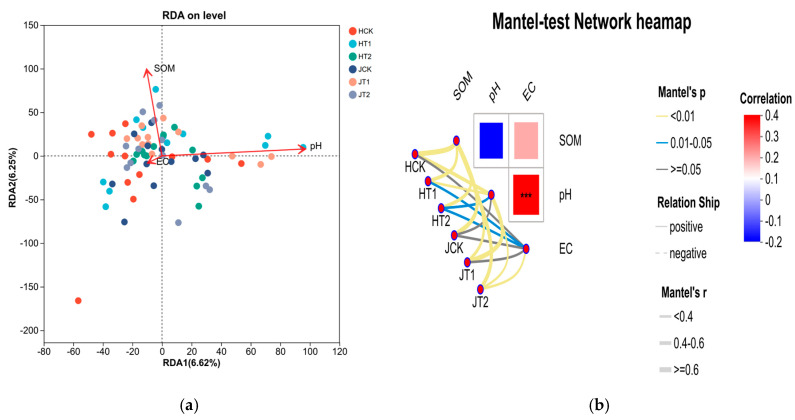
Integrated Association Analysis of Rhizosphere Microbiome, Soil Environment, and Plant Phenotype in Different Sorghum Genotypes. (**a**) RDA ordination showing the influence of soil environmental factors on microbial community structure. (**b**) Mantel test correlation heatmap depicting associations between microbial network modules, soil properties, and plant biomass. Asterisks indicate statistical significance of Mantel’s correlation: *** *p* < 0.001. Color intensity represents the strength of correlation (r value).

RDA and Mantel test results demonstrated that SOM and pH were key drivers of microbial community structure (*p* < 0.05), and biochar-shaped microbial modules were significantly correlated with increased plant biomass (Mantel’s r ≥ 0.5, *p* < 0.05). The RDA ordination plot (Figure 6a) revealed that soil environmental factors significantly influenced the structure of the microbial community. RDA1 and RDA2 explained 6.62% and 6.25% of the total microbial community variation, respectively. SOM and pH were identified as the primary driving factors, with the SOM vector showing a strong positive correlation with RDA2 and the pH vector strongly aligned with RDA1. Samples from different treatment groups exhibited distinct clustering patterns: within the HN16 genotype, HCK samples clustered toward the left, HT2 samples were widely dispersed, and HT1 samples clustered along the positive direction of both SOM and pH. A similar separation was observed among the JCK, JT2, and JT1 samples of the JZN genotype. These patterns indicate that SOM and pH collectively drove the divergence in rhizosphere microbial community structure between HN16 and JZN, and that biochar amendment and salt stress further accentuated this divergence by altering soil conditions.

The Mantel test correlation heatmap (Figure 6b) visually summarized the associations between microbial modules, soil environmental factors (EC, pH, SOM), and plant biomass. Microbial modules in the HT1 group of HN16 showed strong positive correlations with SOM and pH (Mantel’s r ≥ 0.6, *p* < 0.01). Similarly, in the JZN genotype, modules in the JT1 group were strongly associated with SOM (Mantel’s r ≥ 0.6, *p* < 0.01). Further analysis confirmed that these SOM- and pH-linked microbial modules were also significantly positively correlated with plant biomass (Mantel’s r ≥ 0.5, *p* < 0.05), indicating that biochar shaped specific microbial modules that positively influenced plant growth by improving SOM and pH. In contrast, microbial modules in the salt-stressed groups (HT2, JT2) exhibited weaker correlations with environmental factors and plant biomass, reflecting the disruptive effect of salt stress on microbe–environment–plant interactions.

In conclusion, soil EC, pH, and SOM were key factors driving rhizosphere microbial community structure in both HN16 and JZN sorghum genotypes. Biochar strengthened the positive interplay among microbes, soil environment, and plants by improving SOM and pH conditions, thereby fostering microbial modules conducive to plant growth. In contrast, salt stress disrupted this functional balance.

## 4. Discussion

### 4.1. Plant Genotype as the Primary Driver of Rhizosphere Microbiome Assembly

Salt stress fundamentally disrupts soil physicochemical equilibrium, creating an environment hostile to both plants and their associated microbes [23,24]. While the detrimental effects of salinity on soil properties are well-documented, our study reveals a more nuanced picture: biochar’s ability to counteract these effects is strongly modulated by plant genotype. Rather than acting as a universal remedy, biochar’s efficacy in reducing EC, buffering pH, and improving soil structure (Figure 1) depended on whether it was applied to the salt-sensitive HN16 or the salt-tolerant JZN.

These mechanisms allow biochar not only to directly mitigate salt stress by immobilizing salts via its strong adsorption capacity, significantly reducing soil EC (Figure 1c,d), but also to enhance soil water retention and aeration via its high porosity and specific surface area. The core amelioration mechanisms observed in our experiment are closely linked to the physicochemical characteristics of the tested biochar, namely its high iodine adsorption value (992 mg/g), high carbon content (83.1%), and alkaline nature (pH 8.3). Research by Jeffery et al. [25,26] indicated that biochar application can increase soil pH by up to 0.1–2.0 units. In the drylands of northern China, a major wheat-growing region [27], soils are predominantly alkaline. Excessively high soil pH can inhibit enzyme activity and reduce nutrient availability, thereby diminishing plant nutrient uptake and leading to crop yield reduction [28]. In this context, the buffering effect of biochar on soil pH is particularly crucial, as it effectively counteracts potential extreme alkalization induced by salt stress, creating a suitable pH environment for plant roots. In the rhizosphere of the salt-tolerant cultivar JZN, this buffering effect more readily established a micro-domain soil condition closer to neutral, further optimizing the rhizosphere microenvironment.

Regarding soil EC, biochar’s strong adsorption capacity effectively reduced salt stress intensity [29], with genotype-specific efficacy patterns evident in the differential EC reductions (Figure 1c,d). Similarly, improvements in soil structure and organic matter content followed genotype-dependent trajectories (Figure 1e–j), reflecting how biochar’s physical and chemical amendments are processed differently by salt-sensitive versus salt-tolerant cultivars. This not only enhanced soil fertility but also provided ample carbon sources for microbial activity, laying the material foundation for subsequent optimization of the microbial community [30].

### 4.2. Impact of Biochar on the Diversity and Structure of the Sorghum Rhizosphere Microbial Community

Previous studies have established that salt stress suppresses rhizosphere microbial communities, impairing their functional capacity to support plant growth [31]. Our findings corroborate this general pattern but reveal important genotype-specific nuances. While both HN16 and JZN exhibited reduced microbial diversity under salt stress (Figure 2a,c), the recovery trajectories under biochar amendment diverged markedly. JZN, despite showing a more pronounced initial decline, demonstrated a superior capacity to rebuild a diverse and stable microbiome with biochar assistance. This suggests that salt-tolerant genotypes may possess an enhanced ability to leverage soil amendments for microbiome restoration—a trait potentially linked to their differential root exudation patterns and microbial recruitment strategies.

This observation likely reflects the inherent temporal dynamics and compensatory resilience of rhizosphere microbial communities. Transient fluctuations in richness could arise from the rapid, compensatory recruitment of indigenous halotolerant or stress-adapted taxa during early or moderate stress phases, a phenomenon supported by studies on microbial community assembly under perturbation [31]. Crucially, however, sustained salt exposure ultimately resulted in a significant net decline in taxonomic richness over the entire growth period, which aligns with the well-documented suppressive effect of salinity on soil microbial diversity [32]. These dynamics underscore that short-term compensatory responses do not offset the long-term detrimental impact of salt stress, thereby highlighting the essential role of biochar in mitigating this decline in a genotype-dependent manner.

The more pronounced initial decline in species richness observed in JZN (Figure 2a) may represent not vulnerability, but a distinct resilience strategy. While both genotypes experienced diversity loss under salt stress, JZN’s superior recovery trajectory—manifesting as restored diversity (Shannon index) and enhanced network stability (Figure 5)—suggests a pre-adapted capacity to leverage biochar for microbiome restructuring, consistent with its overall salt-tolerant phenotype. Beyond merely providing a habitat through its porous structure, biochar appears to function as a signaling matrix that differentially influences root exudation patterns in a genotype-specific manner. These genotype-specific root exudation patterns, in turn, selectively enrich distinct microbial consortia, as clearly demonstrated by the differential enrichment of key taxa under biochar treatment (Figure 3a,b). For the salt-sensitive HN16, biochar may stimulate the secretion of root exudates that attract rapidly colonizing, stress-mitigating taxa such as *Sporosarcina*; in contrast, for the salt-tolerant JZN, it promotes a more diverse root exudate profile that supports the assembly of a complex, cooperative microbial network centered on *Salinimicrobium*. This genotype-dependent modulation of the “cry for help” hypothesis—where plants recruit beneficial microorganisms via root exudates to cope with environmental stress—warrants further investigation. This mechanism is supported by studies on chemical-mediated plant–microbe interactions in the rhizosphere [33,34,35,36,37,38].

Furthermore, the enrichment of halophilic and extreme halophilic taxa such as *Salinimicrobium* and *Sporosarcina* likely originates from the indigenous soil microbial pool, which includes both bulk soil and resident rhizosphere microbial seed banks [39,40,41]. Under salt stress, biochar-amended soil provides a more stable microhabitat (e.g., improved porosity, moderated pH, and increased organic matter, Figure 1e–j) that favors the proliferation of these stress-adapted taxa. Simultaneously, genotype-specific root exudates act as selective signals, promoting the recruitment of these microbes from the soil reservoir into the rhizosphere niche [10,42]. This two-step process—habitat filtering by biochar and plant-mediated selection—explains how sorghum genotypes assemble distinct functional microbiomes under saline conditions, culminating in the formation of genotype-specific core microbial assemblages (Figure 4) and co-occurrence networks (Figure 5).

Moreover, appropriate amounts of biochar can stimulate microbial metabolic activity, further promoting species richness [32]. Under salt stress conditions, biochar addition effectively reverses the degradation trend of microbial communities. By maintaining their species diversity and structural stability [33], biochar provides crucial micro-ecological support for plant salt resistance. The enhancing effect of biochar on microbial diversity also exhibits genotypic dependence: for JZN, the ACE index under T1 treatment nearly recovered to control levels, and the Shannon index during key growth stages even exceeded the control (Figure 2a,c), indicating that biochar not only alleviated the negative impacts of salt stress but also proactively optimized its rhizosphere microecology. For HN16, the biochar effect primarily manifested as stable maintenance of diversity, without optimization surpassing the control. PCoA analysis showed that the microbial community structure of the T1 group was closer to the CK group, and this “reversion” trend was more obvious in JZN (Figure 2e,f), suggesting that biochar guides the remodeling of the microbial community towards a non-stressed state, with this guiding effect being more significant in the salt-tolerant cultivar. The essence of this differential response lies in the directional regulation of the microbial community by biochar, which enriches stress-resistant functional taxa (Figure 3), strengthens positive synergies among species, and constructs more stable microbial co-occurrence networks (Figure 5) [34]. Our findings align with recent perspectives that plant genotype exerts a deterministic influence on rhizosphere microbiome assembly, modulating both taxonomic composition and functional potential [17,18,22,42,43], as evidenced by the clear separation in community structure between HN16 and JZN even under control conditions (Figure 2d). The genotype-specific recruitment of stress-responsive taxa observed here (Figure 3 and Figure 4) underscores the importance of host genetic background in mediating plant–microbe interactions under abiotic stress. This genotype-driven assembly may be further modulated by biochar, which can act as an ecological filter to enrich beneficial consortia in a host-specific manner, ultimately strengthening the linkages between the optimized soil environment (e.g., SOM and pH, Figure 1), the reshaped microbiome, and improved plant performance, as revealed by integrated association analysis (Figure 6). Concurrently, the increased soil organic matter and optimized pH environment provided by biochar supply ample energy for microorganisms and reduce the inhibition of microbes by extreme pH, further promoting the stability and functional performance of the microbial community, forming a positive feedback loop of “biochar–soil environment–microbial community” [35].

### 4.3. Effects of Biochar on the Microbial Community Under the Dual-Driven Influence of Environment and Genotype

Recent studies highlight that biochar can reshape microbial networks by enhancing cooperative interactions and stabilizing community structure [19,20]. In our study, the enrichment of taxa such as *Pontibacter* (often associated with organic matter decomposition) and *Deinococcus* (known for stress resilience) under salt stress suggests a functional restructuring of the microbiome toward nutrient cycling and stability maintenance. This aligns with the emerging view that biochar fosters ecologically coherent microbial modules that support plant resilience under abiotic stress [19].

Microorganisms are pivotal to the soil ecosystem, with their community structure directly influencing soil health and crop growth. The application of soil amendments can directionally regulate the soil micro-environment by altering microbial habitats and metabolic activities [36]. Biochar-mediated regulation of the soil microbial community structure demonstrates significant environmental dependence and genotype specificity. Its mechanism of action is associated not only with alterations in soil physicochemical properties [37] but is also directionally driven by the innate characteristics of plant cultivars. Zhang et al. [38] observed that in arsenic-contaminated soil, biochar addition significantly modified microbial community composition through multiple pathways, including increased soil organic carbon content, improved permeability, and pH regulation. This significantly reduced the relative abundance of Actinobacteria and specifically decreased the proportion of *Nocardioidaceae*. Given that both Actinobacteria and its subordinate *Nocardioidaceae* are Gram-positive bacteria, the reduction in these groups directly led to a decline in Gram-positive bacterial biomass within the soil [39,40,41], ultimately triggering a comprehensive shift in microbial community structure. Furthermore, pH serves as a key determinant of bacterial taxonomic richness and composition; its modification by biochar further intensified this community restructuring process [42].

The genotype-specific microbial restructuring induced by biochar under salt stress reveals fundamentally divergent plant–microbe interaction strategies. While both cultivars assembled distinct core microbiomes even under control conditions (Figure 2d and Figure 4)—a testament to the deterministic role of plant genotype—their response to biochar amendment followed contrasting trajectories [43]. HN16’s association with stress-mitigating taxa like Bacillus and Arthrobacter suggests a defensive strategy focused on immediate stress alleviation. In contrast, JZN’s enrichment of decomposers and secondary metabolite producers (*Sphingomonas*, *Streptomyces*) indicates a more proactive approach aimed at long-term soil conditioning and systemic resilience. This dichotomy extends beyond taxonomy to ecological function: HN16 appears to prioritize survival under acute stress, while JZN invests in building a cooperative microbial network capable of sustaining growth under persistent salinity. Our observation that HN16 and JZN assembled distinct core microbiomes aligns with the established principle that plant genotype dominates rhizosphere assembly patterns [17,22]. The biochar-amended, genotype-specific co-occurrence networks we observed—characterized by enhanced modularity and positive interactions—resemble the stabilized and cooperative microbial networks reported in other biochar-amended systems [20,21]. Furthermore, the link between these biochar-shaped microbial modules and increased soil organic matter (SOM) in our study echoes findings that connect microbial community restructuring with improved soil fertility [19].

Biochar’s regulation operates through a genotype-specific lens, reinforcing rather than overriding host-controlled assembly patterns. HN16’s enrichment of early-colonizing stress mitigators (e.g., *Sporosarcina*) aligns with an acute defense strategy, while JZN’s sustained enrichment of specialized taxa and modular network optimization (Figure 3 and Figure 5) reflects a proactive, system-level resilience approach. These divergent trajectories highlight how biochar amplifies pre-existing genotype-specific plant–microbe interaction strategies [43]. This, coupled with long-term synergies from improved soil structure and enhanced fertility, enabled a more comprehensive strategy for stress resistance and growth promotion, reflecting a more robust and integrated response of the salt-tolerant cultivar to biochar application.

## 5. Conclusions

Moving beyond its established role in soil conditioning, this study unveils a novel mechanism whereby biochar serves as a genotype-specific microbiome modulator under salt stress. We demonstrate that the ameliorative effect of biochar is principally mediated through the host-selective assembly of beneficial microbial consortia, a process that diverges fundamentally between salt-sensitive and salt-tolerant sorghum genotypes. This discovery shifts the paradigm from viewing biochar as a universal amendment to recognizing it as a precision tool for rhizosphere engineering. Its importance to sustainable agriculture lies in providing a mechanistic basis for developing crop-specific biochar formulations and management protocols, thereby enhancing salinity resilience while reducing input waste. To translate this insight into practice, future efforts must prioritize integrating plant genotype data into biochar recommendation systems and breeding crops that better leverage microbiome-mediated stress resistance.

Biochar acts as a precise ecological regulator of the soil microbiome. It not only reversed the salt stress–induced decline in microbial species richness but also facilitated the assembly of customized, salt-resistant microbial consortia through the selective enrichment of genotype–specific functional taxa. This regulatory effect exhibited significant differences between the two sorghum cultivars with contrasting salt tolerance: The salt-sensitive HN16 showed a rapid enrichment of salt-tolerant and nitrogen-fixing taxa, represented by *g_Sporosarcina*, during the early growth stages, suggesting a strategy for acute stress mitigation. In contrast, the salt-tolerant JZN, by persistently enriching multi-functional taxa and forming a tightly structured, highly modular co-occurrence network, fully activated the ‘system-optimizing’ potential of biochar. This led to structural upgrading and functional improvement of the microbial network, thereby establishing a systematic long-term resistance mechanism and realizing synergistic gains in sustained stress resistance and growth promotion.

Thus, plant genotype emerged as the core factor determining the pathway and efficacy of biochar amelioration. Based on their long-term evolutionary history shaping distinct rhizosphere microecological characteristics, different cultivars exhibited fundamentally different response strategies to biochar input: HN16 primarily leveraged the ‘stress-buffering’ function of biochar, rapidly reducing salt damage to maintain basic growth. Conversely, JZN fully activated the ‘system-optimizing’ potential of biochar, achieving structural upgrading and functional improvement of the microbial network, consequently realizing synergistic gains in sustained stress resistance and growth promotion. This genotype-rooted specific response mechanism fundamentally explains the differential amelioration effects observed from the same biochar treatment applied to different sorghum cultivars.

While this study elucidates the genotype-specific assembly of rhizosphere microbiomes, the underlying molecular mechanisms and causal relationships remain to be fully deciphered. Future research should employ metatranscriptomics to verify the functional activity of the recruited taxa and track the dynamics of root exudate profiles under biochar amendment. Additionally, cross-inoculation experiments could disentangle the relative contributions of plant genotype versus soil legacy effects in shaping these adaptive microbiomes. Ultimately, harnessing genotype-specific plant–microbe interactions, as elucidated here, provides a mechanistic pathway to promote sustainable agricultural practices. Optimizing biochar applications tailored to crop genotype can enhance salinity resilience, paving the way for developing effective strategies for plant–microbiome management in the new crops era.

## Figures and Tables

**Table 1:** Changes in soil composition of different treatment groups.

Treatment	Description	Biochar Content (g kg^−1^)	NaCl Content (g kg^−1^)	Growth Period
CK	Control (no stress, no biochar)	0	0	S0, S1, S2, S3, S4
T1	Salt stress with biochar amendment	20	5	S0, S1, S2, S3, S4
T2	Salt stress only (no biochar)	0	5	S0, S1, S2, S3, S4

## Data Availability

The raw data supporting the conclusions of this article will be made available by the authors on request.

## Abbreviations

The following abbreviations are used in this manuscript:

HN16	salt-sensitive cultivar Henong 16
JZN	salt-tolerant cultivar Jizaonuo No. 1
NaCl	sodium chloride
CK	Control check
T1	Contains 5‰ NaCl and 2% biochar
T2	Contains 5‰ NaCl
HCK	HN16 Control check
HT1	HN16 Contains 5‰ NaCl and 2% biochar
HT2	HN16 Contains 5‰ NaCl
JCK	JZN Control check
JT1	JZN Contains 5‰ NaCl and 2% biochar
JT2	JZN Contains 5‰ NaCl
EC	Soil electrical conductivity
pH	Potential of Hydrogen
SOM	Soil organic matter
SBD	Soil bulk density
SP	Soil porosity

## Appendix A

**Table A1:** Names and Sources of 185 Sorghum Varieties.

Number	Name	Source
1	Zaoshu 1	Baoding, China
2	Zaoshu 2	Baoding, China
3	Zaoshu 5	Baoding, China
4	Zaoshu 8	Baoding, China
5	Zaoshu 12	Baoding, China
6	Zaoshu 13	Baoding, China
7	Zaoshu 16	Baoding, China
8	Zaoshu 17	Baoding, China
9	Zaoshu 18	Baoding, China
10	Tianza 1	Baoding, China
11	Tian 2	Baoding, China
12	Tian 4	Baoding, China
13	Tian 5	Baoding, China
14	Tian 8	Baoding, China
15	Tian 9	Baoding, China
16	Tian 10	Baoding, China
17	Tian 11	Baoding, China
18	Tian 115	Baoding, China
19	Tian 133	Baoding, China
20	Tian 138	Baoding, China
21	Tian 139	Baoding, China
22	Tian 151	Baoding, China
23	Tian 319	Baoding, China
24	MY1	Beijing, China
25	MY2	Beijing, China
26	MY3	Beijing, China
27	MY4	Beijing, China
28	MY5	Beijing, China
29	MY6	Beijing, China
30	MY9	Beijing, China
31	MY10	Beijing, China
32	MY11	Beijing, China
33	MY12	Beijing, China
34	MY13	Beijing, China
35	MY14	Beijing, China
36	MY15	Beijing, China
37	MY16	Beijing, China
38	MY17	Beijing, China
39	MY18	Beijing, China
40	MY19	Beijing, China
41	MY20	Beijing, China
42	MY21	Beijing, China
43	MY22	Beijing, China
44	Hongyin No.1	Baoding, China
45	Hongyin No.2	Baoding, China
46	Hong No.2	Baoding, China
47	Hongyin No.3	Baoding, China
48	Hongmaonuo No.6	Baoding, China
49	Sorghum Sweet Stalk	Baoding, China
50	Sanchisan	Baoding, China
51	Sticky Sorghum	Baoding, China
52	Xiaobairen	Baoding, China
53	Chunlei	Baoding, China
54	Chunyuan	Baoding, China
55	Heike	Baoding, China
56	Silimei	Baoding, China
57	Jinfu No.1	Baoding, China
58	Xingxiangliang No.2	Baoding, China
59	Xin 52	Baoding, China
60	Ying 654	Baoding, China
61	Broom-use 2	Baoding, China
62	Cangzhou Broom-use	Baoding, China
63	Henan Broom-use	Baoding, China
64	Tian 6	Baoding, China
65	Hai 1	Baoding, China
66	Hai 2	Baoding, China
67	Hai 3	Baoding, China
68	Hai 4	Baoding, China
69	Hai 5	Baoding, China
70	Sweet Stalk-1	Baoding, China
71	Sweet Stalk-2	Baoding, China
72	Zhang 2	Baoding, China
73	10B	Baoding, China
74	cp90107	Baoding, China
75	cp90155	Baoding, China
76	B45	Baoding, China
77	K4	Baoding, China
78	R19	Baoding, China
79	H8B	Baoding, China
80	HM65	Baoding, China
81	Sudan 1 Original	Baoding, China
82	Sudan 1-1	Baoding, China
83	Sudan 1-2	Baoding, China
84	90111	Baoding, China
85	Sudan Grass	Baoding, China
86	Sudan 3 Original	Baoding, China
87	Sudan 4-1	Baoding, China
88	Sudan 4-2	Baoding, China
89	Jizaonuo No.1	Baoding, China
90	4 (8)	Baoding, China
91	Kangya B	Baoding, China
92	6 (4)	Baoding, China
93	Huangluosa	Bin County, China
94	Xin 4B	China
95	South Africa	South Africa
96	Sugarcane Seed Sorghum	Zhen’an, China
97	GLZ	Baoding, China
98	Qisiwu Sorghum	Qihe, China
99	Vespa	Germany
100	94CW5045	China
101	Heikufen Sorghum	Lingqiu, China
102	IS-3977	India
103	Swaziland	Eswatini
104	Xiaohongke	Jiaocheng, China
105	Malawi	Malawi
106	South Africa	South Africa
107	Loose Panicle Sweet Sorghum	Jinzhou Academy of Agricultural Sciences, China
108	Daqingmi	Balin Left Banner, China
109	HQ1	Baoding, China
110	HQ2	Baoding, China
111	QN35	Baoding, China
112	407/Hongyingzi	Baoding, China
113	407/Jiqing’aihong	Baoding, China
114	Jiqing’aihong	Baoding, China
115	Zimbabwe	Zimbabwe
116	Kangsi	Cangzhou Bureau of Agriculture, China
117	Qiansan	Baoding, China
118	Luoziji	Changle, China
119	Waibozhang	Tongliao, China
120	Dahongpao	Ziyang, China
121	Huangnian Sorghum	Qinhuangdao, China
122	Heitian Sorghum	Ningshan, China
123	Songgai	China
124	Hongyingzi	Baoding, China
125	IS-2248	India
126	L407B	China
127	ICSV739	India
128	MN-839	USA
129	Nigeria	Nigeria
130	Changtingzi 9	Heilongjiang, China
131	Hongliuzi	Cangshan, China
132	South Africa	South Africa
133	Ethiopia	Ethiopia
134	Pingdingxiang	Bayan, China
135	Muhui 54-2	Mudanjiang, China
136	Huangniuwe	Lan County, China
137	Sheyan	China
138	Changtingganz	huanghe, China
139	Tieshamao	Xiangyuan, China
140	L407B	China
141	R71,30241	China
142	Tieshamao	Mexico
143	Tieshamao	China
144	936815-16	China
145	A-3194	India
146	Sweet Sorghum	Qichun, China
147	Dawaitou	Anze, China
148	Dawaitou	Anze, China
149	Hong Sorghum	Lufeng, China
150	Huangkeben	Funing, China
151	Huangniangao	Ba County, China
152	K131	China
153	LR62	China
154	Yidu Rice Sorghum	Changwei Academy of Agricultural Sciences, China
155	91CC515	China
156	Liantang’ai	Guiyang, China
157	United States of America	USA
158	Lesotho	Lesotho
159	0-5	India
160	United States of America	USA
161	Swaziland	Eswatini
162	M20	Baoding, China
163	M201	Baoding, China
164	M648	Baoding, China
165	A504(80R)	India
166	Henong 16	Baoding, China
167	Ping 41B	China
168	ICSH10	India
169	30278	China
170	EMS	Baoding, China
171	407B	Baoding, China
172	Dasheyan	Laoting, China
173	6-5A	Baoding, China
174	Guandongqing	Laoting, China
175	Tiehui 154	China
176	Lesotho	Lesotho
177	8.5222B	India
178	6-5B	Baoding, China
179	M27	Baoding, China
180	M37	Baoding, China
181	M26	Baoding, China
182	M44	Baoding, China
183	M45	Baoding, China
184	M50	Baoding, China
185	M614	Baoding, China
